# Analysis of Competing Risks of Causes of Death in Cancer Patients from Golestan, Iran over Twelve Years (2004-2016)

**DOI:** 10.31557/APJCP.2021.22.10.3137

**Published:** 2021-10

**Authors:** Mostafa Zare, Susan Hasanpour-Heidari, Shahryar Semnani, Abdolreza Fazel, Seyed Mehdi Sedaghat, Yasamin Semnani, Seyed Mohsen Mansuri, Masoomeh Gholami, Marzieh Araghi, Gholamreza Roshandel

**Affiliations:** 1 *Golestan Research Center of Gastroenterology and Hepatology, Golestan University of Medical Sciences, Gorgan, Iran.*; 2 *Omid Cancer Research Center, Omid Preventive Medicine and Heath Promotion Center, Golestan University of Medical Sciences, Gorgan, Iran. *; 3 *Cancer Research Center, Golestan University of Medical Sciences, Gorgan, Iran. *; 4 *Deputy of Public Health, Golestan University of Medical Sciences, Gorgan, Iran.*; 5 *Statistics and Information Technology Office, Golestan University of Medical Sciences, Gorgan, Iran. *; 6 *Death Registry Unit, Deputy of Public Health, Golestan University of Medical Sciences, Gorgan, Iran. *; 7 *Department of Epidemiology and Biostatistics, School of Public Health, Imperial College London, London, UK. *

**Keywords:** Cancer, mortality, cause of death, Golestan, Iran

## Abstract

**Background:**

Cancer-related causes of death (cancer CoD) are the main etiologies of death in cancer patients. Recent increase in survival rates of cancer patients resulted in higher risk of dying from causes other than cancer, called competing causes of death (competing CoD). We aim to characterize competing CoD among cancer patients in Golestan province, Northern Iran.

**Methods:**

Data on cancer incidence was obtained from the Golestan population-based cancer registry (GPCR) dataset. Data on causes of death was obtained from the Golestan death registry (GDR) dataset. Using a linkage method between the GPCR and GDR dataset, we prepared the study dataset including data on vital status and causes of death in our cancer patients. The proportions of cancer CoD and competing CoD were calculated. Multivariate logistic regression analysis was considered to assess the relationship between competing CoD and other variables.

**Results:**

Overall, 4,184 cancer patients died in the study population, including 2,488 men (59.9%). Cause of death in 3,455 cases was cancer and 729 cases (17.4%) died due to competing CoD. Ischemic heart disease (40.7%) was the most common competing CoD in our population. Higher survival rate was the strongest variable related to the competing CoD (adjusted OR=1.91; 95%CI: 1.61-2.26). Residence area, age group and year of death were other indicators of competing CoD in our population.

**Conclusion:**

Our results suggest high rates of competing CoD in our cancer patients. Competing CoD should be mentioned in cancer control planning both in clinical practice as well as in public health policy making.

## Introduction

Cancer is a major health problem with an estimated number of 18 million incident cases and 10 million deaths in 2018 worldwide (Bray et al., 2018). Ongoing advances in cancer treatments, early detection and screening programs resulted in decreasing trends of mortality in cancer patients. It is projected that by the year 2040, there will be 26 million cancer survivor especially for those ages 60 and over (Shapiro, 2018). Cancer-related causes of death (cancer CoD) are the main etiologies of death in cancer patients. In addition, cancer survivors may also be at the risk of dying from causes other than cancer, called competing causes of death (competing CoD) (Massa et al., 2017). Competing CoD are associated with risk factors (competing risk) that compete with the cancer to cause death in cancer patients (Varadhan et al., 2010). Massa et al reported that nearly one in three head and neck cancer patients dies from competing causes of death including cardiovascular, lung and liver diseases (Massa et al., 2017). Information on competing risks and competing CoD enables clinicians and policy makers to effectively manage cancer patients (Kanitkar et al., 2018).

The Golestan province in Northern Iran has nearly 2 million population, known as a high-risk area for upper gastrointestinal cancers (Kmet and Mahboubi, 1972; Mahboubi et al., 1973). Recent reports suggested increasing in 1-, 3- and 5-year survival rate in esophageal cancer patients from this region (Golalipour et al., 2017). Improvement in access to healthcare facilities and consequently increase in survival rate may suggest competing CoD as an important issue in management of cancer patients in this high-risk population. All these, make it important to investigate the causes of death, and specifically, the competing CoD during these 12 years.

## Materials and Methods

This cross-section study was conducted on cancer patients from Golestan province of Iran who died between 2004 and 2016. We obtained data on cancer incidence from the Golestan population-based cancer registry (GPCR) dataset (Roshandel et al., 2012; Roshandel et al., 2018). The GPCR, a voting member of the international association of cancer registries (IACR), is a high-quality cancer registry collecting data on newly diagnosed primary cancer patients through Golestan province considering standard protocols and guidelines developed by the international agency for research on cancer (IARC). Details on data collection, data process and analysis of the GPCR have been described previously (Roshandel et al., 2018). The GPCR used both International Classification of Disease for Oncology-3rd edition (ICD-O-3) (Fritz et al, 2013) and the International Statistical Classification of Disease and Related Health Problems (ICD-10) (World Health Organization, 2004) for coding tumor characteristics. 

Data on causes of death were obtained from the Golestan death registry (GDR) dataset (Hasanpour-Heidari et al., 2019). The GDR secretariat is located in the Deputy of Heath of Golestan University of Medical Sciences and is responsible for registering information on death in Golestan province using an official death certificate. The GDR used the International Statistical Classification of Disease and Related Health Problems (ICD-10) (Jafari et al., 2009; Organization, 2011) for coding causes of death.

Following data quality check, the GPCR and the GDR datasets were linked to identify data on mortality and causes of death in cancer patients using an in-house health data linkage software developed by the GPCR secretariat. After completing the linkage process, all cancer patients who died during the study period were selected and entered into the analysis. The cancer sites were categorized using ICD-10 coding system (supplementary Table 1). 

Causes of death were categorized at two levels. At level 1, we categorized causes of death into two major groups including cancer-related causes of death (cancer CoD) and competing causes of death (competing CoD). Then at level 2, the competing CoD were further categorized into 13 groups considering their frequencies and according to the ICD-10 system (supplementary Table 2). 

The proportions of competing CoD were calculated by sex (male vs female), age groups, residence area (urban vs rural), year of death and survival rate. According to World Health Organization (WHO) definition for elderly population and mean age of our study subjects (60), our participants were categorized into <60 and >=60 years old. Based on year of death, the study subjects were categorized into two time periods including the first period (2004-2009) and the second period (2010-2016). Considering patients’ median survival rate, we categorized the study participants into two groups including those with survival rate of lower than 5.5 months and those with survival rate of equal or higher than 5.5 months. 

The relationship between competing CoD and grouping variables were assessed using multivariate logistic regression analysis. Crude and adjusted odds ratios (ORs) with their 95% confidence intervals (95% CI) were calculated. SPSS v16 was used for statistical analysis. P-values of less than 0.05 were considered as significant.

The study protocol was approved by ethics committee of the Iranian National Institute for Medical Research Development (NIMAD) (code: IR.NIMAD.REC.1397.117).

## Results

Overall, 4,184 cancer patients died in Golestan province during 2004-2016, including 2,488 men (59.9%) and 1,696 women (40.5%). The mean (SD) and median (IQR) of participants’ age were 59.91(17.6) and 62 (50-73) years, respectively. Residential distribution showed that 1,946 (46.5%) of cancer death cases lived in urban and 2,238 (53.5%) lived in rural area. Table 1 shows the distribution of cancer patients who died during 2004-2016 in Golestan province, by cancer site. It shows that Gastrointestinal (GI) tract cancers had the highest incidence between cancer death cases. While female genitalia, GI tract and brain had highest proportion among cancer CoD cases, cancer cases with urine tract (29.3%), skin, bone and soft tissue (28.6%) and male genitalia (23.8%) had greater competing CoD, respectively.

**Figure 1:**
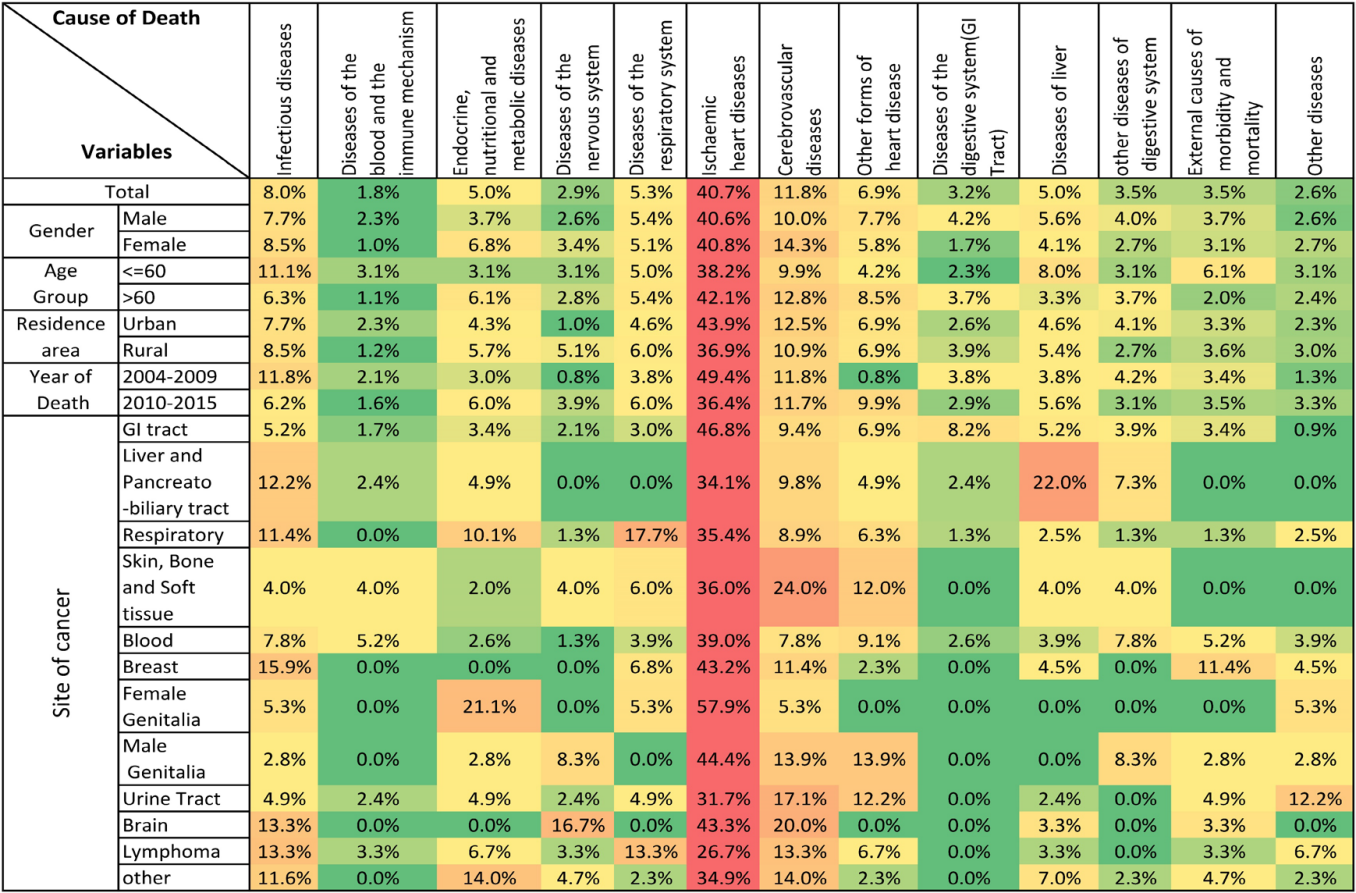
Distribution of Competing Causes of Death by Different Variables in Cancer Patients from Golestan Province, Iran, 2004-2016 (in each row, the red and green colors represent highest and lowest proportions, respectively)

Among 4,184 cancer patients who died during the study period, the cause of death in 3,455 cases was Cancer CoD and 729 (17.4%) died due to competing CoD. In Six of 729 cases the CoD could not be defined. For the remaining 723 cancer cases a specific competing CoD was defined and these cases were entered into the final analysis. The result shows that 434 (59.5%) are men with mean (SD) age of 65.57 (16.47) and 295 (40.5%) are women with mean (SD) age of 61.98 (17.19). 395(54.2%) cases lived in urban and 334(45.8%) lived in rural areas. The median of survival rate was calculated 5.5 months. Table 2 illustrates that cancer cases with more than 60 years old had higher (20.8%) competing CoD, significantly. In residence area, cancer patients in urban area had significantly higher competing CoD (20.3%) than rural area (14.9%). Cancer patients who died during the latter time period (2010-2016) had significant higher competing CoD (19.5%). Cancer patients with more than 5.5 months of survival rate had significant higher competing CoD (22.2%) than patient with less than 5.5 months of survival rate (13%). Figure 1 shows the distribution of competing CoD by different variables. It suggests that ischemic heart diseases are leading competing CoD in all subgroups of variables such as gender, age group, residence area, year of death and site of cancer. In the earlier years of the study (2004-2009) ischemic heart disease had highest rate of competing CoD (49.4%), while among cancer cases with lymphoma ischemic heart disease had lowest rate of competing CoD (26.7%). In some cancer patients, non-cancer diseases of the cancer site were the most common competing CoD after ischemic heart disease. For example, non-cancer diseases of liver and pancreas were the second common competing CoD (22%) in patients with cancer of the liver and pancreato-biliary tract. Similarly, respiratory diseases were the second common competing CoD (17.7%) in patients with respiratory system cancers. 

**Table 1:** Distribution of Cancer Patients who Died during 2004-2016 in Golestan Province, by Causes of Death (CoD)

Cancer site	All Cancer Death cases Number	Cancer-related COD Number (%)	Competing COD Number (%)
GI tract (Esophagus, Stomach, small intestine, colorectal)	1638	1405 (85.8%)	233 (14.2%)
Liver and Pancreato-biliary tract	271	230 (84.9%)	41 (15.1%)
Respiratory	405	324 (80.0%)	81 (20.0%)
Skin, Bone and Soft tissue	175	125 (71.4%)	50 (28.6%)
Leukemia	492	415 (84.3%)	77 (15.7%)
Breast	276	232 (84.1%)	44 (15.9%)
Female Genitalia	139	120 (86.3%)	19 (13.7%)
Male Genitalia	151	115 (76.2%)	36 (23.8%)
Urinary Tract	150	106 (70.7%)	44 (29.3%)
Brain	209	178 (85.2%)	31 (14.8%)
Lymphoma	133	103 (77.4%)	30 (22.6%)
other	145	102 (70.3%)	43 (29.7%)

**Table 2:** Predictors of Competing Cause of Death (CoD) in Cancer Cases in Golestan Province, Iran (2004-2016)

Variables		Number of Competing CoD/Total	Proportion of Competing CoD	Un-adjusted		Adjusted	
				OR (95% CI)	P-value	OR (95% CI)	P-value
Gender	Female	295/1696	17.40%	Ref	0.97	Ref	0.9
	Male	434/2488	17.40%	1 (0.85-1.17)		1.01 (0.35-1.20)	
Age Group	<=60	265/1949	13.60%	Ref	<0.01	Ref	<0.01
	>60	464/2235	20.80%	1.66 (1.41-1.96)		1.34 (1.13-1.58)	
Residence area	Rural	334/2238	14.90%	Ref	<0.01	Ref	<0.01
	Urban	395/1946	20.30%	1.45 (1.24-1.70)		1.72 (1.45-2.04)	
Year of Death	2004-2009	238/1663	14.30%	Ref	<0.01	Ref	<0.01
	2010-2016	491/2521	19.50%	1.45 (1.22-1.71)		1.28 (1.08-1.53)	
Survival time (month)	<5.5	263/2027	13.00%	Ref	<0.01	Ref	<0.01
	>=5.5	452/2038	22.20%	1.91 (1.62-2.26)		1.91 (1.61-2.26)	

OR, odds ratio; CI, confidence interval

## Discussion

The current study focused on non-cancer causes of death among cancer patients by linking population-based cancer registry with death registry data. Our findings illustrate competing CoD in patients with cancer has important aspect that should be considered toward non-cancer mortality risks among cancer patients. Cancer mortality patterns help policy makers to prioritize healthcare resources.

Our result showed that 17.4% of cancer deaths occurred due to competing CoD. There have been few studies about reporting CoD among cancer patients in other countries. The study in the South Korea reported CoD among cancer patients diagnosed from 2000 to 2016, showed 9.8% of death among cancer patients belonged to the non-cancer CoD.(Oh et al., 2020) Another study from the United States, indicated that almost 5.2% of cancer patients death was due to non-cancer CoD among cancer patient who diagnosed from 1973 to 2012 (Zaorsky et al., 2017). Higher rate of competing CoD in our population may be partly explained by differences in types of most common cancers (Oh et al., 2020). Patients with fatal cancers (with lower survival rates) will more possibly die due to the underlying malignant disease (cancer CoD), while those with non-fatal cancers will survive longer and may be at higher risk of dying due to competing CoD. Recent reports from Golestan suggested considerable increasing trends in incidence of non-fatal cancers (e.g. breast and colorectal cancers), while the trends of fatal cancers (e.g. esophageal and stomach cancers) were decreasing (Roshandel et al., 2020). Therefore, high incidence rates and the increasing trends of non-fatal cancers may partly explain high proportion of competing CoD in Golestan population. Quality of diagnostic and therapeutic services, registration systems and finally quality of death registry data may also be considered as other possible explanations for high proportion of competing CoD in our population (German et al., 2011; Dinkelspiel et al., 2015; Hasanpour-Heidari et al., 2019).

Ischemic heart disease (40.7%) was the most common competing CoD in our population. Previous studies also suggested ischemic heart diseases as the leading competing CoD among cancer patients compared with general population (Song et al., 2019; Oh et al., 2020). Ageing is a mutual risk factor for both cancer and ischemic heart disease (Barrett-Connor et al., 1984; Shammas, 2011) therefore, higher rates of ischemic heart disease in elderly may partly explain the higher rates of ischemic heart disease as a competing CoD in cancer patients. Moreover, long term use of cancer-specific therapies (e.g. chemotherapy) may increase the risk for cardiaotoxity and will consequently increase the risk of cardiovascular diseases and cardiovascular mortalities in cancer patients (Patnaik et al., 2011; Curigliano et al., 2016; Abdel-Qadir et al., 2017; Abdel-Rahman, 2017; Sturgeon et al., 2019). Therefore, patients with diagnosed cancer are at high risk for cardiovascular disease and should be followed up with cardiologist along with oncologist (Pushparaji et al., 2020; Stoltzfus et al., 2020).

Patients with skin, bone and soft tissue, urinary tract and male genitalia cancers had higher rates of competing CoD. Our results also suggested lowest rates of competing CoD in patients with female genitalia, GI tract and brain cancer. The study in 2014 from the United States concluded that competing CoD for patients with skin, bone and soft tissue cancers have been increased due to the fact that early cancer treatment had major effect on competing CoD among those patients and prevent disease recurrence (Youn et al., 2014). Other studies have showed that women diagnosed with female genitalia cancers (e.g ovarian, endometrium) have substantial risk of cancer CoD (Ward et al., 2012; Dinkelspiel et al., 2015). Some cancers among female genitalia (e.g ovarian cancer) could have the risk of second malignancy so it can be contributed to high cancer CoD in this population (Zaorsky et al., 2017). Fatality of cancers and availability of healthcare services for screening and treatment of cancers may affect survival in cancer patients and consequently the rates of competing CoD (Dinkelspiel et al., 2015).

According to our results the most common competing CoD in patients with female genitalia cancer is cardiovascular disease. In 2012, a study from United States concluded that cardiovascular disease could be the leading CoD among female with endometrial cancer (Ward et al., 2012). Women with female genitalia cancer have mutual risk factors with heart disease such as obesity and metabolic syndrome, which can lead to higher to cardiovascular death among these population (Ward et al., 2012; Dinkelspiel et al., 2015; Laskey et al., 2016; Felix et al., 2017). Therefore, it is suggested to follow patients with female genitalia cancers for cardiovascular comorbidities.

Our result also represents that competing CoD was significantly higher in cancer cases older than 60 years when compared to younger age groups. Co-morbidities may occur and threaten elderly people in addition to cancer. In the study of cause of death among elderly patients in western Iran demonstrated that circulatory system disease, infectious disease, and the respiratory system disease had the highest rates of cause of death among patients with the age of 60 years old and more, respectively (Siabani et al., 2020). 

Based on our results competing CoD in urban area was more than rural area. Rural-urban disparities can be addressed by the obstacles to access healthcare services such as screening, early detection and treatment facilities in rural area in comparison with urban residents (Blake et al., 2017; Garcia et al., 2017). Therefore, cancer patients could be followed up in better conditions in urban rather than rural area, resulting in better survival and higher rates of competing CoD due to other co-morbidities. 

Regarding the time period, the risk of competing cause of death was significantly higher during the latter period in comparison with the earlier years. This may be due to the fact that the quality of death registry for accuracy and completeness had been improved (Hasanpour-Heidari et al., 2019) and another reason may attribute to the longevity and better survival in cancer patients due to better access to effective healthcare services during the second period, resulting in higher rates of competing CoD (Botta et al., 2019). 

In this study, we used high-quality cancer incidence data from the Golestan population-based cancer registry over a long period. Quality of cancer data and its large sample size were the most important advances of our study. Poor quality of death registry data, especially in earlier years of the study period was a limitation of the presents study. This point should be considered in interpretation of or findings. 

In conclusion, our results suggested high rates of competing CoD in our cancer patients. We found the ischemic heart disease as the most common competing CoD in our population. Higher survival rate, living in urban area and older age group were the strongest variables related to the risk of competing CoD in our study. Competing CoD should be considered by clinicians in management of disease in cancer patients. It should also be mentioned in cancer control planning in public health policy making.

## Author Contribution Statement

MZ: collaborated in data collection and processing; wrote the manuscript; SH-H: collaborated in data analysis, wrote the manuscript; SS, AF: initiated, conceptualized and designed the study; edited and critically reviewed manuscript; SMS, YS: collaborated in collection of data; critically reviewed manuscript; SMM, MG: collaborated in data processing and quality control; critically reviewed manuscript; MA: edited and critically reviewed manuscript; collaborated in interpretation of results; GR: conceptualized and designed the study; performed statistical analysis; wrote the manuscript; All authors read and approved the final manuscript.
